# Wellen’s Syndrome: A Rare Case

**DOI:** 10.7759/cureus.25158

**Published:** 2022-05-20

**Authors:** Govind Nagdev, Gajanan Chavan, Gaurang M Aurangabadkar

**Affiliations:** 1 Emergency Medicine, Jawaharlal Nehru Medical College, Wardha, IND; 2 Respiratory Medicine, Jawaharlal Nehru Medical College, Wardha, IND

**Keywords:** wellen’s syndrome, coronary artery angiography, left circumflex artery (lcx), electrocardiography (ecg), 2d echocardiography

## Abstract

Wellen’s syndrome is associated with critical stenosis of the left anterior descending coronary artery. Based on the electrocardiography (ECG) pattern, Wellen’s syndrome can be classified into type 1 (deeply inverted T-waves, mainly in lead V2 and V3) or type 2 (biphasic T-waves). T-wave abnormalities are often also found in V1 and V4 and rarely in V5 and V6. The pattern of ECG changes correlates with proximal left anterior descending artery stenosis. This characteristic ECG pattern is a sign of impending myocardial infarction and is equivalent to ST-elevated myocardial infarction. Often, these subtle findings in ECG get misinterpreted or the severity associated with this goes unrecognized. Hence, for emergency physicians, it is important to recognize such uncharacteristic ECG changes for better and timely management of patients. We present this case of Wellen’s pattern in which the coronary lesion was in the left circumflex coronary artery, right coronary artery, and diagonal-1.

## Introduction

Wellen’s syndrome describes a form of abnormal T-waves in the leads V2 and V3 associated with left anterior descending artery (LAD) stenosis. Approximately 75% of patients with these findings have deeply inverted T-waves, and about 25% show a variant form of biphasic T-waves in similar leads [1]. It is also called LAD T-wave syndrome [2]. T-wave abnormalities are often also seen in V1 and V4 and on rare occasions in V5 and V6.

Patients with Wellen’s syndrome are usually pain-free when they present to the emergency department; however, in our case, the patient was concerned about his intense chest pain and wished to get evaluated. Usually, cardiac enzymes are normal or only slightly elevated in patients with Wellen’s syndrome. Electrocardiography (ECG) changes are generally seen when patients are pain-free and may normalize when pain recurs. To aid in detecting changes, ECG can be repeated when pain resolves [3]. Wellen’s syndrome is seen in 15% of patients presenting with unstable angina. Because patients with Wellen’s syndrome are at high risk of critical coronary stenosis and the potential for acute myocardial infarction (MI), they should receive early coronary intervention [4]. The distinctive ECG signs diagnostic of Wellen’s syndrome can help with decision-making before a serious MI occurs [5-7].

Here, we present a case of Wellen’s syndrome in which the patient presented to the emergency department in a pain-free period. His ECG changes were minimal, enzymes were not raised, two-dimensional echocardiography (2D-Echo) showed no regional wall motion abnormalities (RWMA), and the patient had triple vessel disease. This case emphasizes the significance of such alterations and their capacity to reveal the location of severe stenosis.

## Case presentation

**Figure 1:**
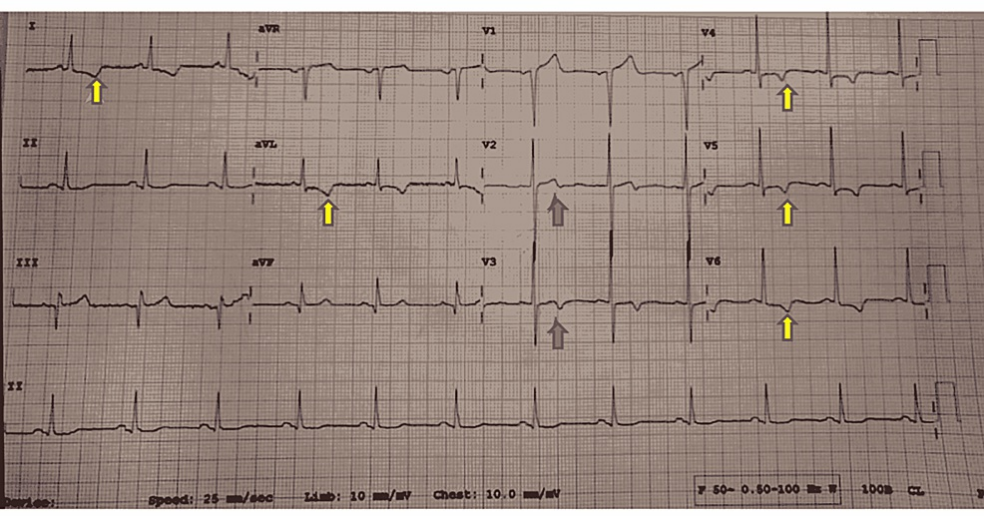
Biphasic T-waves in lead V2 and V3 (red arrows) (typical of type 1 Wellen’s syndrome) and symmetrically inverted T-waves in lead 1, aVL, V4-V6 (yellow arrows) (typical of type 2 Wellen’s syndrome) and Q-waves in lead 3 and aVF.

A 57-year-old man presented to our hospital with chief complaints of chest pain and uneasiness with palpitations and sweating the previous night. He had chest pain again around two to three hours before his presentation to our hospital, which increased by minimal exercise and reduced by rest. The patient was pain-free when he presented to the emergency department. On presentation, an ECG was taken and cardiac enzymes were sent for evaluation. With regards to his medical history, he was a known case of systemic hypertension for eight years on regular antihypertensive medication telmisartan 40 mg once a day. The patient gave no history of any cardiac symptoms in the past. He was a smoker and used to smoke a pack of cigarettes per day for 10-12 years which he stopped eight years back. His father aged 79 years had coronary artery disease and had undergone percutaneous coronary intervention (PCI) six years back. ECG revealed biphasic T-waves in lead V2 and V3 and symmetrically inverted T-waves in lead 1, aVL, V4, V5, and V6, and Q-waves in lead 3 and aVF (Figure 1).

The ECG and clinical criteria for Wellen’s syndrome are presented in Table 1.

**Table 1:** Electrocardiographic and clinical criteria for Wellen’s syndrome. Adapted from de Zwaan et al. [3].

Electrocardiographic and clinical criteria
Deeply inverted and biphasic T-waves in V2 and V3 and on rare occasions in V1, V4, V5, and V6
Normal or slightly elevated cardiac markers
Normal or slight elevation in ST-segment (<1 mm)
Pathological precordial Q-wave absent
Normal R-wave progression
Electrocardiographic changes are seen when pain is absent
A history of angina

The initial coronary angiography findings are illustrated in Table 2.

**Table 2:** Coronary angiography findings post-admission. LCA: left coronary artery; LAD: left anterior descending artery; LCx: left circumflex coronary artery; RCA: right coronary artery

	Findings
LCA	Normal
LAD	Mid LAD 50% stenosed
Diagonal-1	85–90% stenosed
LCx	100% stenosed
RCA	75–80% stenosed

**Figure 2:**
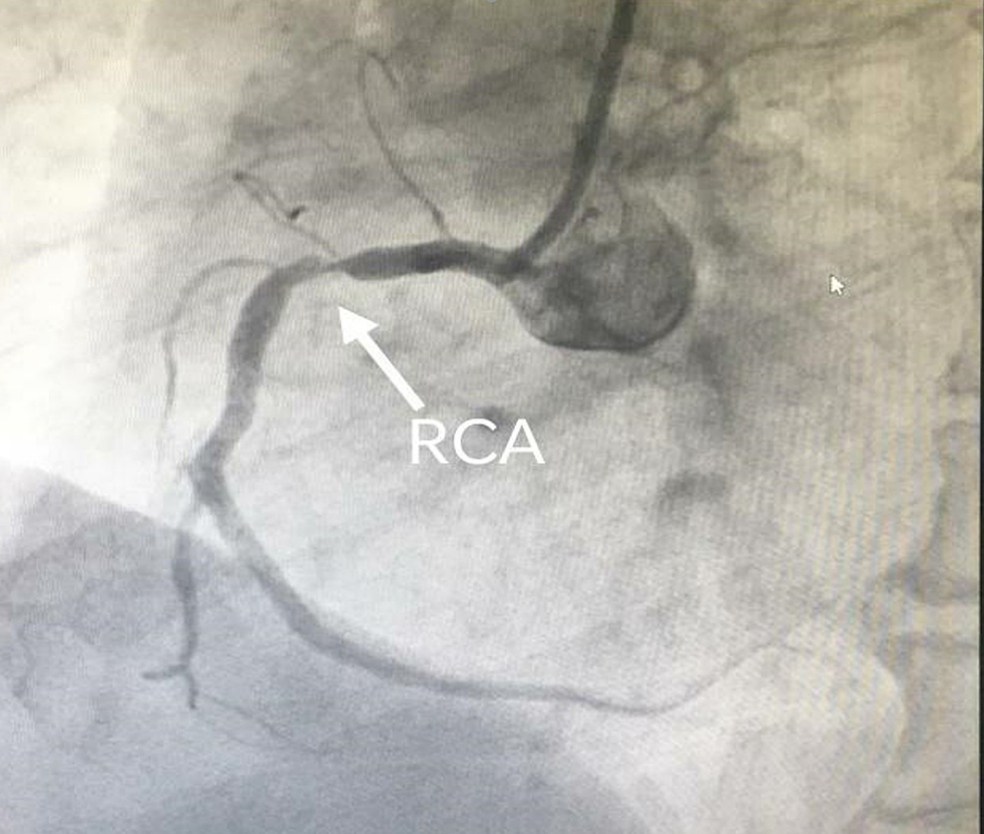
Coronary angiography showing evidence of critical stenosis (80%) in the right coronary artery territory. RCA: right coronary artery

**Figure 3:**
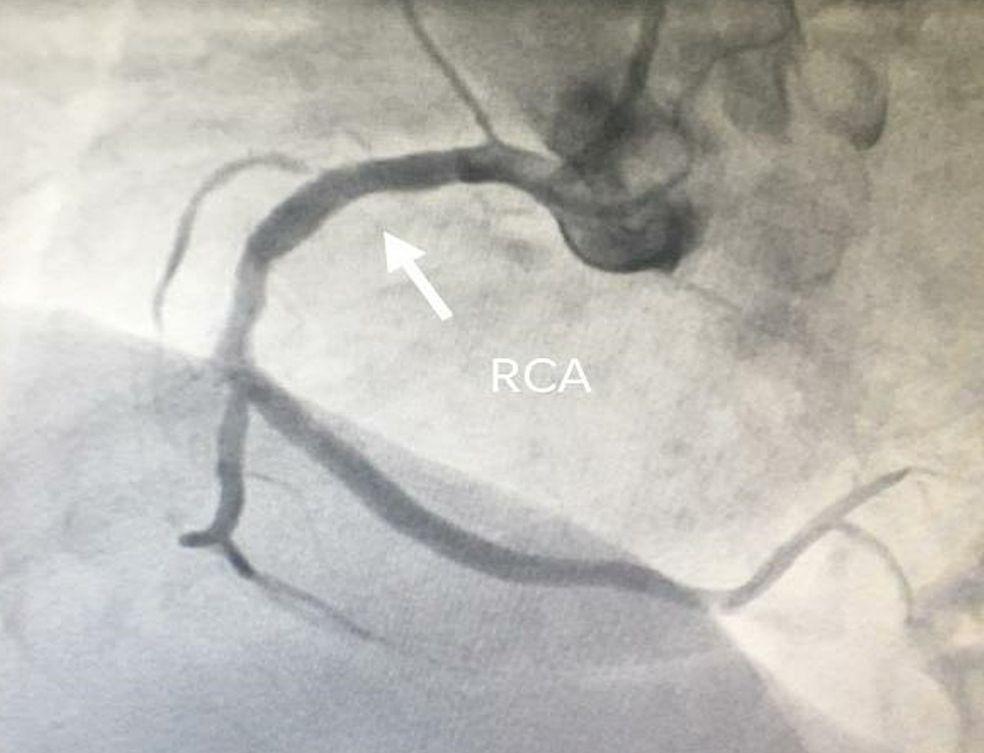
Coronary angiography post-stenting to the right coronary artery. RCA: right coronary artery

The vitals of the patient were within the normal range. General and systemic examination was normal. After 10 minutes of presenting to the emergency department, he again developed chest pain. A standard treatment regimen including aspirin, statin, and ticagrelor along with conventional heparin 3,500 IU intravenously was administered. Within a short span, the pain subsided but ECG changes were persistent. Troponin I HS (quantitative) 18.9 ng/L (normal range upper limit: 19 ng/L). All routine investigations were normal. ECG revealed no RWMA with mild left ventricular hypertrophy, grade 1 diastolic dysfunction, and preserved ejection fraction. Day 2 ECG showed T-wave inversion in lateral leads (1, aVL, V5, V6) and Q-waves in inferior leads (3 and aVF), following which coronary angiography was done. Angiography revealed stenosis of the right coronary artery (RCA) (75-80%) (Figure 2), left circumflex (100%), and D1 (85-90%). A stent to the RCA and balloon angioplasty to diagonal-1 were done without tackling LCx (Figure 3). The patient recovered without complications and was discharged.

## Discussion

Myocardial ischemia typically produces inverted T-waves which are symmetrical and narrow; however, ischemia may also show biphasic T-waves [8]. de Zwaan and colleagues have emphasized the significance of both forms of alterations in T-waves and explained the ECG and clinical criteria [9]. This pattern of clinical and ECG abnormalities is noteworthy and specifically related to proximal LAD stenosis, commonly referred to as Wellen’s syndrome [2,4]. In the original study [2], out of 145 consecutive patients with unstable angina, 26 had a typical ECG pattern of Wellen’s syndrome, and 12 patients who did not undergo coronary revascularization developed extensive anterior wall infarction within a few weeks of admission. Thus, in the emergency department, it is crucial to detect ECG changes suggestive of Wellen’s syndrome at the earliest. Two types of ECG changes have been documented for Wellen’s syndrome. Relatively uncommon biphasic T-waves in V2 and V3 are characterized as type 1. Deeply inverted T-waves in V2 and V3 leads are another prevalent pattern characterized as type 2; on rare occasions, it can be seen in V1, V4, and V5-V6 leads. These ECG changes can appear in pain-free intervals or during chest pain and may become normal or lead to ST-segment elevation. The cause of ECG variations is unknown. Changes can be temporary, last for months, or disappear with treatment.

Coronary angiography of the patient revealed critical stenosis of the RCA (80%), LCx (100%), and diagonal-1 (90%). Despite the fact that the ECG changes in our patient were consistent with those of Wellen’s syndrome, critical stenosis was found in the LCx, RCA, and diagonal-1 instead of the proximal LAD. In addition, mid-LAD was also stenosed (40-50%). PCI to RCA with drug-eluting stent along with balloon angioplasty to diagonal-1 was done without tackling the LCx. The patient recovered without complications and was discharged.

## Conclusions

The significance of biphasic T-waves is often overlooked in emergency rooms, with such findings typically being recorded as not exact or general ECG abnormalities. Finally, despite our patient’s ECG results being characteristic of Wellen’s syndrome, the crucial lesion was discovered in the LCx, RCA, and diagonal-1. This case emphasizes the significance of such alterations and their capability to identify the location of severe stenosis.

## References

[1] Hsu YC, Hsu CW, Chen TC (2017). Type B Wellens' syndrome: electrocardiogram patterns that clinicians should be aware of. Ci Ji Yi Xue Za Zhi.

[2] Aufderheide TP, Gibler WB (1998). Acute ischemic coronary syndromes. Rosen's Emergency Medicine: Concepts and Clinical Practice.

[3] de Zwaan C, Bär FW, Janssen JH (1989). Angiographic and clinical characteristics of patients with unstable angina showing an ECG pattern indicating critical narrowing of the proximal LAD coronary artery. Am Heart J.

[4] Rhinehardt J, Brady WJ, Perron AD, Mattu A (2002). Electrocardiographic manifestations of Wellens' syndrome. Am J Emerg Med.

[5] Tandy TK, Bottomy DP, Lewis JG (1999). Wellens' syndrome. Ann Emerg Med.

[6] Ayer A, Terkelsen CJ (2014). Difficult ECGs in STEMI: lessons learned from serial sampling of pre- and in-hospital ECGs. J Electrocardiol.

[7] Raheja P, Sekhar A, Lewis D, Samson R, Sardana V, Coram R (2017). Wellens' syndrome over the past three decades. J Cardiovasc Med (Hagerstown).

[8] Liu M, Han C, Ke J (2016). Wellenoid T-wave is an important indicator for severe coronary artery stenosis. Int J Cardiol.

[9] de Zwaan C, Bär FW, Wellens HJ (1982). Characteristic electrocardiographic pattern indicating a critical stenosis high in left anterior descending coronary artery in patients admitted because of impending myocardial infarction. Am Heart J.

